# Optical Coherence Tomography as a Biomarker for Differential Diagnostics in Nystagmus: Ganglion Cell Layer Thickness Ratio

**DOI:** 10.3390/jcm11174941

**Published:** 2022-08-23

**Authors:** Khaldoon O. Al-Nosairy, Elisabeth V. Quanz, Julia Biermann, Michael B. Hoffmann

**Affiliations:** 1 Department of Ophthalmology, Faculty of Medicine, Otto-von-Guericke University, 39120 Magdeburg, Germany; 2 Department of Ophthalmology, University of Muenster Medical Centre, 48149 Muenster, Germany; 3 Center for Behavioral Brain Sciences, 39118 Magdeburg, Germany

**Keywords:** nystagmus, GCLT quotient, OCT, macular thickness asymmetry, misrouting, albinism, foveal hypoplasia, retina

## Abstract

In albinism, with the use of optical coherence tomography (OCT), a thinning of the macular ganglion cell layer was recently reported. As a consequence, the relevant OCT measure, i.e., a reduction of the temporal/nasal ganglion cell layer thickness quotient (GCLTQ), is a strong candidate for a novel biomarker of albinism. However, nystagmus is a common trait in albinism and is known as a potential confound of imaging techniques. Therefore, there is a need to determine the impact of nystagmus without albinism on the GCLTQ. In this bi-center study, the retinal GCLTQ was determined (OCT Spectralis, Heidelberg Engineering, Heidelberg, Germany) for healthy controls (*n* = 5, 10 eyes) vs. participants with nystagmus and albinism (N_albinism_, *n* = 8, 15 eyes), and with nystagmus of other origins (N_other_, *n* = 11, 17 eyes). Macular OCT with 25 horizontal B scans 20 × 20° with 9 automated real time tracking (ART) frames centered on the retina was obtained for each group. From the sectoral GCLTs of the early treatment diabetic retinopathy study (ETDRS) circular thickness maps, i.e., 3 mm and 6 mm ETDRS rings, GCLTQ I and GCLTQ II were determined. Both GCLTQs were reduced in N_albinism_ (GCLTQ I and II: 0.78 and 0.77, *p* < 0.001) compared to N_other_ (0.91 and 0.93) and healthy controls (0.89 and 0.95). The discrimination of N_albinism_ from N_other_ via GCLTQ I and II had an area under the curve of 80 and 82% with an optimal cutoff point of 0.86 and 0.88, respectively. In conclusion, lower GCLTQ in N_albinism_ appears as a distinguished feature in albinism-related nystagmus as opposed to other causes of nystagmus.

## 1. Introduction

Optical coherence tomography (OCT) provides non-invasive surrogate structural biomarkers that aid diagnosis and/or further understanding of various ophthalmological diseases. Albinism, for example, demonstrates characteristic structural retinal abnormalities, i.e., foveal hypoplasia (FH), where OCT holds promising potentials as a defining biomarker [1].

Albinism, a rare inherited disorder, affects hair, skin, and eyes, i.e., oculocutaneous albinism, or eyes only, i.e., ocular albinism [2]. In fact, albinism demonstrates several ophthalmic characteristics including nystagmus, iris translucency, and optic nerve fibers’ misrouting at the optic chiasm [3]. Further, albinism shows inter-subject variability in the typical ocular features and this variability can overlap with other diagnoses associated with the infantile nystagmus syndrome (INS) [3]. Actually, there is no single defining feature in albinism, as highlighted in a recent investigation of more than 500 participants with albinism [3], where no single diagnostic test had 100% penetrance. Even misrouting, determined by visual evoked potential (VEP), was not detected in 16% of the above patient cohort [3]. In the present study, OCT was additionally employed to address this heterogeneity and the limited availability of molecular diagnosis in albinism.

The use of OCT has been suggested for the identification of FH in albinism [4,5,6]. However, this is not straightforward, as FH may be absent in albinism and present in other diseases than albinism [3] or may occur without any disease status [7]. In fact, more detailed quantitative OCT assessments might offer better insights into retinal characterizations in these disease entities. A stimulating macular OCT finding in this respect was the report of the thinning of the temporal ganglion cell layer thickness (GCLT), and hence a reduction of the temporal/nasal GCLT quotient (GCLTQ) in albinism-related FH compared to other causes of FH and to healthy controls [1]. In fact, this OCT approach of macular GCLT assessment might be of assistance to establish a novel diagnostic biomarker in albinism [1].

Importantly, nystagmus is a common trait of albinism. Nystagmus is known, however, to have the potential to interfere with imaging measures. It is therefore critical to assess whether the above GCLTQ reduction is exclusive to albinism-related nystagmus or whether it might be more generally associated with nystagmus, especially in the absence of FH. In this study, we therefore aimed to (i) investigate and compare GCLTQ in nystagmus participants driven by albinism vs. other etiologies and (ii) to estimate the area under the curve (AUC) of GCLTQ as a diagnostic biomarker in albinism vs. nystagmus of other origins. In the present study, we propose that the GCLTQ reduction, previously observed in albinism, is a novel OCT biomarker specific to albinism as opposed to other causes of nystagmus.

## 2. Materials and Methods

This bi-center study adhered to the tenets of the Declaration of Helsinki and the Ethical Committee of the Otto-von-Guericke University of Magdeburg and Muenster approved the study protocol. The consents of two participants from the Magdeburg cohort were utilized with the standard diagnostic procedure performed in the clinic.

### 2.1. Participants

In this bi-centric study, participants were recruited from the university eye hospitals in Magdeburg and Münster into three groups: (a) healthy controls (HC; *n* = 5; mean age (range) [y]: 37 (21–56)), i.e., logMAR ≤ 0 best corrected visual acuity; (b) participants with nystagmus and without misrouting of the optic nerves (N_other_; *n* = 11; 7 acquired, 4 with infantile nystagmus syndrome (INS); (mean age range) [y]: 27 (8–56)); and (c) participants with nystagmus and misrouting of the optic nerves (N_albinism_; *n* = 8; (mean age range) [y]: 19 (5–52)) diagnosed as albinism after Kruijt et al. [3], i.e., each of 3 major criteria: (I) FH ≥ grade 2, (II) negative VEP correlations, and (III) ocular hypopigmentation, i.e., iris translucency or fundus hypopigmentation, or 2 major and 2 minor criteria, i.e., minor criteria are: (i) nystagmus, (ii) FH grade 1, (iii) fundus hypopigmentation grade 1, and (iv) skin and hair hypopigmentation, i.e., lighter than that of their siblings and parents.

FH was classified after Thomas et al. [8] into grades: G1, absence of plexiform layers’ extrusion; G2, G1 plus absence of the foveal pit; G3, G2 plus the absence of outer segment lengthening; and G4, G3 plus absence of outer nuclear layer lengthening.

Fundus hypopigmentation was graded after Schmitz et al. [9] as follows into grades: G1, peripheral retinal hypopigmentation; G2, distinct G1 plus central hypopigmentation; G3, strongly pronounced G2 (macular and foveal hypoplasia); and G4, G3 plus atypical choroideremia. The included participants of the present study are detailed in Table 1.

Exclusion criteria were epilepsy, dizziness, diabetic retinopathy, and neurological diseases unrelated to nystagmus. There were no significant differences of the mean age between groups (healthy vs. N_other_ vs. N_albinism_ group (mean age): 37 vs. 27 vs. 19; *p* = 0.12). The BCVA was significantly lowest in the latter group, i.e., 0.4 logMAR, while it was the highest for the healthy group, i.e., −0.15 logMAR; see Table 2.

### 2.2. Procedure and Measurements

All participants underwent complete ophthalmic examinations. Visual fields were tested where necessary utilizing a Humphrey Field Analyzer 3 (Carl Zeiss Meditec AG, Jena, Germany; Swedish Interactive Threshold Algorithm 24–2 protocol (SITA Faster)).

#### 2.2.1. Visual Evoked Potentials

Three scalp electrodes (Oz, O1, O2) were placed and referenced to the Fz (according to the international 10–20 System) [10]. Following the International Society for Clinical Electrophysiology of Vision (ISCEV) standards for VEP recordings [11], the EP2000 evoked potential system was used for stimulation, recording, and analysis of pattern onset–offset VEP in all but four participants of the Magdeburg cohort [12] and in one participant of the Münster cohort. The stimuli were presented on a monochrome CRT monitor (MDG403, Philips; P45 phosphor; 75 Hz) in a dimly lit room. The stimulation and recording were set up according to the EP2000 testing paradigm which included a pattern onset epoch of 40 ms and 468 ms offset presenting 3 check sizes, i.e., 0.5°, 1°, and 2° with a spaced-averaged mean luminance of 50 cd/m^2^ and contrast of 100% at a viewing distance of 114 cm. The VEP signals were connected to an amplifier (Grass Model 15, Astro-Med Inc., West Warwick, RI, USA), 100,000 times amplified, and band pass filtered (0.3, 70 Hz). VEP trials with a threshold of 90 µV were online rejected. Two VEP blocks were monocularly recorded and later averaged using IGOR (IGOR Pro, WaveMetrics, Portland, OR, USA). VEPs were digitally filtered (high-pass and low-pass cutoff: 0 and 40 Hz) and averaged using IGOR. To determine the presence of misrouting, the difference of the averaged VEP over the opposing hemisphere were calculated for each eye’s recording. Subsequently, the correlation of the interhemispheric difference traces for the two eyes was determined via the correlation coefficient [13]. Albinotic misrouting of the optic nerves typically results in negative correlation coefficients in albinism between right and left eyes difference traces [12].

#### 2.2.2. Optical Coherence Tomography (OCT)

The OCT for the macula was performed with 25 horizontal B scans 20° × 20° volume scans centered on the retina with 9 automated real time tracking (ART) frames with the Spectral domain OCT (OCT Spectralis, Heidelberg Engineering, Heidelberg, Germany). FH as well as GCLTQ were graded and calculated after Thomas et al. as detailed above [8]. Based on the macular scan, GCLT was computed using the inbuilt multilayer segmentation algorithm of the Heidelberg OCT device. GCLTs within the two nasal and two temporal quadrants of the ETDRS areas established by the Early Treatment Diabetic Retinopathy study were exported to calculate temporal/nasal GCLTQ [1]. GCLTQ I and II are the ratios within the 3 mm and 6 mm ETDRS rings, respectively.

### 2.3. Statistics

Further analysis for VEP and OCT was conducted using the programs IGOR and R [14]. The Shapiro–Wilk test was used to check the normality of the data and parametric or nonparametric tests were applied accordingly. *p* values were adjusted with the Sidak correction for multiple testing. The area under the curve (AUC) of the receiver operating characteristics was also calculated for GCLTQ I and II to differentiate between eyes with albinism and other eyes. Sample size was calculated based on Brücher et al. [1] where an area under the curve of 89% of ganglion cell thickness ratio II between the albinism and non-albinism groups with a power of 95% and *p* value < 0.05 mandated the inclusion of 10 eyes per group.

## 3. Results

### 3.1. Overview of Ophthalmological Characteristics

As expected, the presence of iris transillumination defects, fundus hypopigmentation, and FH were significantly prevalent in N_albinism_ compared to the other two groups (Fisher’s exact tests *p* ≤ 0.001). N_albinism_ displayed the only eyes with iris translucency (*n* = 8/15) and fundus hypopigmentation (*n* = 13) (Grade 3 (*n* = 7), Grade 4 (*n* = 6)). Further, FH was more likely associated with N_albinism_ (*n* = 15/15) (Grade 1 (*n* = 1), Grade 3 (*n* = 10), Grade 4 (*n* = 4)) while only two eyes (*n* = 2/17) (Grade 1 (*n* = 1) and Grade 4 (*n* = 1) from two separate individuals) were affected in N_other_.

### 3.2. OCT Analysis: GCLTQ

**Figure 1:**
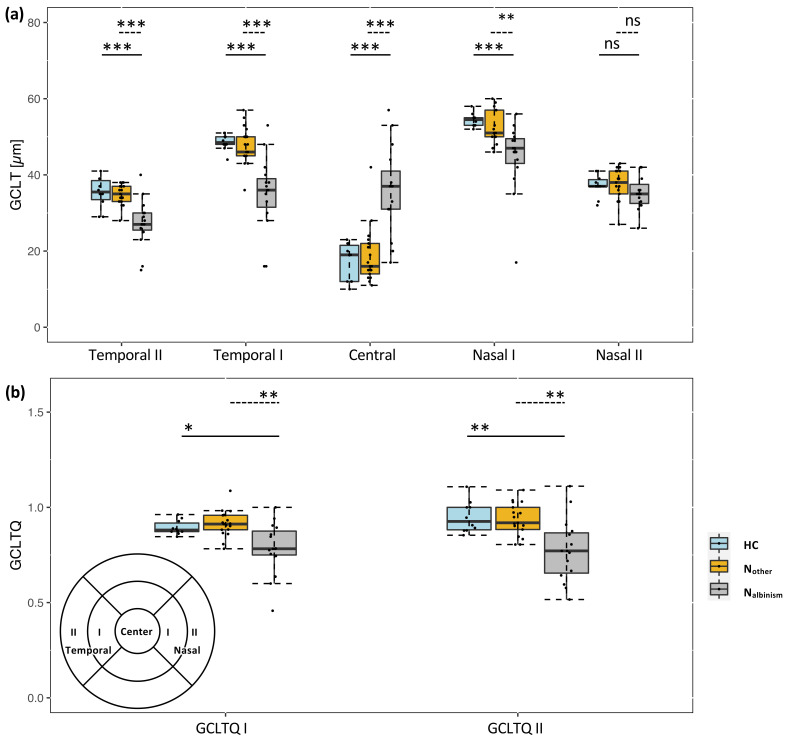
Paracentral ganglion cell layer thickness (GCLT) and quotient (GCLTQ) analysis. (**a**) Comparison of the GCLT in the 5 relevant sectors within the ETDRS macular OCT scan for healthy (HC) vs. albinism (N_albinism_) and other causes of nystagmus (N_other_). (**b**) Group comparisons of the GCLT quotients (GCLTQs) of temporal to nasal quadrants within the 3 mm macular ETDRS ring, i.e., GCLTQ I, and the outer 6 mm ring, i.e., GCLTQ II. Lower left panel is the ETDRS scan layout with areas of interest. *p* values were corrected after Sidak. * *p* < 0.05; ** *p* < 0.01; *** *p* < 0.0001. ns = non-significant *p* value.

The GCLT was significantly different between the albinism and the reference groups for the N1, T1, central, and T2 quadrants; see Figure 1a. The central GCLT was, in accordance with the prevalence of FH, significantly thicker in the N_albinism_ group than the other two groups (*p* < 0.001). In contrast, the paracentral GCTL was significantly reduced in N_albinism_ for the quadrants N1, T1, and T2 compared to healthy and N_other_. There is, in accordance with previous records [1,15], an asymmetry of the pericentral thinning of the GCLT in albinism, which is reflected by the GCLTQ as reported previously. The GCLTQ I was statistically lower (*p* = 0.001) in the N_albinism_ group, i.e., a mean of 0.78, vs. healthy and N_other_, i.e., 0.89 and 0.91, respectively. Likewise, GCLTQ II showed the same trend (*p* < 0.001), i.e., 0.77 vs. 0.95 and 0.93, respectively; see Figure 1b.

### 3.3. GCLTQ Diagnostic Performance

In order to establish the sensitivity of this OCT metric to detect albinism-related macular changes from other groups, we determined the cut-off points for GCLT I and II ratios and their respective AUC. The calculated optimal cutoff points were 0.857 and 0.875, respectively. The GCLT I ratio had an AUC of 80% with a sensitivity and specificity of 73% and 89% at the determined cutoff point, respectively, and the GCLT II ratio showed a comparable performance, i.e., 82% AUC with 80% and 81% at the optimal cutoff point, respectively.

## 4. Discussion

We tested whether the OCT finding of decreased GCLTQ, which was previously reported for albinism, is independent of the presence of nystagmus, a condition that is common to albinism. Here, we confirmed previous findings of decreased GCLTQ in albinism and extended this finding by demonstrating normal GCLTQ for a critical control group, i.e., patients with nystagmus in the absence of albinism and (except for two of the patients) without FH. This strongly suggests that GCLTQ reduction is indeed specific to albinism and that it might be employed as a diagnostic metric in albinism independent from the presence of nystagmus.

Stimulated by a previous incidental finding in a Japanese family [16], Brücher et al. and Woertz et al. [1,15] reported a thinning of temporal paracentral GCLT in albinism with FH vs. other causes of FH. Brücher et al. [1] set a cut-off of the temporal/nasal GCLTQ to identify FH with albinism where GCLTQ II is more sensitive than GCLTQ I, 0.72 vs. 0.80, respectively. In the present study, we observed a similar trend, but with slightly higher optimal cut-off points, i.e., of 0.88 vs. 0.86 for GCLTQ II and I, respectively, in albinism with nystagmus vs. other groups. Further, Brücher et al. [1] reported somewhat higher AUCs (89% and 91%) than our present study (80% and 82%) for GCLTQ I and II, respectively. Both studies have in common that the sensitivity was highest for GCLTQ II than I (Brücher et al. [1]: 100% vs. 90%; present study: 80% vs. 73%). Taken together, the discrimination performance in the present study appears slightly inferior to the preceding study, which is likely associated with the different non-albinotic reference group in the present study, i.e., N_other_.

Beyond its clinical relevance, the finding of temporal thinning of macular GCLT raises the question of the (i) underlying mechanisms and (ii) its relevance to the post-retinal visual pathway architecture in albinism [17,18,19,20]. (i) The origin of the GCLT thinning in albinism might be related to the severity of retinal changes in albinism, especially FH, a typical trait of albinism. For this purpose, future studies on datasets with greater sample sizes are needed to perform meaningful correlation analyses of the relationship of temporal GCLT thinning and the grade of FH. (ii) Normally the line of decussation, which separates the retinal ganglion cells that project to the opposite hemisphere from those that remain ipsilateral, coincides with the fovea. In albinism, the misrouting of the optic nerves at the optic chiasm leads to a shift of the line of decussation into the temporal retina [21], and thus substantially more axons cross the midline. The size of this lateral shift affects on average 8 degrees in the visual field, but has been reported to vary between 2 and 15 degrees of visual angle, underlining a large interindividual variability in albinism [13,21]. This variability is to some degree related to the severity of the pigmentation deficit of the affected individuals [22]. It is generally assumed and supported by experimental evidence [2] that the projection error of part of the temporal retinal ganglion cells is related to changes in the retinal physiology and development and its inter-individual variability. It is currently unclear whether there is a link between the GCLTQ reduction in albinism and the degree of optic nerve misrouting. This prompts the question of whether the severity of retinal changes, as reflected by the GCLTQ reduction in albinism, is related to changes in the post-retinal pathways, e.g., the degree of misrouting of the optic nerves. Uncovering these relations is likely to assist uncovering the nature and origin of the changes of the visual pathway architecture in albinism. Datasets with greater sample sizes are needed to perform the necessary correlation analyses. Therefore, the publication of datasets from these rare patients [23] is highly desirable, as these can be merged subsequently for the required analyses.

Although the present study is unique by its investigation of GCLTQ in groups with nystagmus with and without albinism, the following limitations deserve attention: (i) There was a comparatively small sample size; however, the rarity of patients with albinism or nystagmus made it difficult to include more. (ii) The long scanning time required to acquire meaningful OCT data led to the exclusion of a number of patients as they had fixation that was too unstable. As the technical evolution of retinal OCT is proceeding rapidly, faster scanning times and optimized eye tracking will hopefully lead to an extension of explorable patients in future.

In conclusion, the present study lends further support of selective thinning of the paracentral temporal retina specifically in albinism. While this might serve as a biomarker to aid in the identification of albinism, it might also help to uncover the impact of these retinal changes on the post-retinal visual pathways in albinism.

## Figures and Tables

**Table 1:** Participants’ grouping and key features.

Center-ID	Eye	Group	Nystagmus Type	BCVA OD/OS	Fundus Hypopigmentation OD/OS	Misrouting VEP	Foveal Hypoplasia OD/OS
MD-BWZ133	Both	HC	None	−0.1/−0.2	0/0	−	0/0
MD-KZJ780	Both	HC	None	−0.1/−0.1	0/0	−	0/0
MD-LHP483	Both	HC	None	0/0	0/0	−	0/0
MD-RKM968	Both	HC	None	−0.2/−0.2	0/0	−	0/0
MD-YHW227	Both	HC	None	−0.2/−0.2	0/0	−	0/0
MD-BJA815	OS	INS	J/H	0.2/0.2	0/0	−	n.a./0
MD-ENH995	Both	INS	J/H	0.6/0.49	0/0	−	0/0
MD-JDG458	Both	INS	P/H	0.3/0.3	0/0	−	0/0
MD-MFY773	OD	INS	J/H	0.1/0.3	0/0	−	0/n.a.
MD-PEP763	Both	INS	J/H	0/0.4	0/0	−	0/0
MD-SUQ660	Both	INS	J/H	−0.1/−0.1	0/0	−	0/0
MD-WQE170	OD	INS	P/H	0.2/0.2	0/0	−	1/n.a.
MS-MS01	OS	INS	J/H	0.1/0.3	0/0	n.a.	n.a./0
MS-MS02	OD	INS	J/H	0.4/0.4	0/0	n.a.	4/n.a.
MD-TGY248	Both	AN	J/V	0/0	0/0	−	0/0
MD-TIO945	Both	AN	J/H	0/−0.1	0/0	−	0/0
MD-HAA059	Both	Albinism ⁎	n.a. ‡	0.6/0.7	3/3	+	3/4
MD-JTE807	Both	Albinism	J/H	0.4/0.8	0/0	+	4/4
MD-NLE254	Both	Albinism ⁎	n.a. ‡	0.4/0.4	2/2	+	3/3
MD-PYV946	OS	Albinism	J/H	0.4/0.4	2/2	+	n.a./1
MD-TCU787	Both	Albinism ⁎	n.a. ‡	0.8/0.6	2/2	+	3/3
MS-MS03	Both	Albinism	J/H	0.5/0.4	2/2	n.a.	3/3
MS-MS04	Both	Albinism	P/H	0.4/0.4	3/3	+	3/3
MS-MS05	Both	Albinism ⁎	J/H	0.5/0.4	3/3	n.a.	3/4

MD/MS: Magdeburg/Muenster cohort; OD: right eye; OS: left eye; BCVA [logMAR]: best corrected visual acuity; VEP: misrouting visual evoked potential; “+”/“−” indicates presence/absence of optic nerve (negative/positive correlation coefficient between both eyes’ inter-hemispherical activation difference); INS: idiopathic infantile syndrome (excluding albinism); HC: healthy control; AN: acquired nystagmus (causes: Arnold-Chiari syndrome, pons bleeding, hydrocephalus shunt operation); J/H: jerk horizontal nystagmus; P/H: pendular horizontal nystagmus; J/V: jerk vertical nystagmus; “n.a.”: was not assessed; ‡ nystagmus diagnosis was made by qualified ophthalmologist. Albinism diagnosis: according to Kruijt et al.’s criteria [3], as detailed in Materials and Methods together with the grading schemes. ⁎ Indicates the presence of iris translucency.

**Table 2:** Studied groups’ characteristics.

	HC	N_other_	N_albinism_				
				*p*	HC vs. N_other_	HC vs. N_albinism_	N_other_ vs. N_albinism_
*n* (f)	5 (3)	11 (6)	8 (5)				
Age [y]	37 (21–56)	27 (8–56)	19 (5–52)	0.12			
Eyes (OD)	10 (5)	17 (9)	15 (7)	1.0§			
SE [Diopters] ‡	[−0.25 (−2–+3.4)]	[−1.0 (−7–+3.5)]	[1.5 (−4.4–+6.3)]	**0.005** ‡	0.16	0.07	**0.001**
BCVA [LogMAR] ‡	[−0.15 (0–−0.20)]	[0.20 (+1–−0.1)]	[0.4 (+0.8–+0.4)]	**<0.001** ‡	**<0.001**	**<0.001**	**<0.001**
**(*n* of Eyes)** **Ophthalmological** **examination**	**10**	**17**	**15**				
Strabismus	0	2	4	0.16 §			
Iris translucency	0	0	8	**<0.001** §			
Fundus hypopigmentation				**<0.001** §			
0	10	17	2				
1	0	0	0				
2	0	0	7				
3	0	0	6				
**OCT**							
FH				**<0.001** §			
0	10	15	0				
1	0	1	1				
2	0	0	0				
3	0	0	10				
4	0	1	4				
**Retinal GCLT [µm]**							
Nasal II	37 ± 3	37 ± 4	35 ± 5	0.31			
Nasal I ‡	[55 (52–58)]	[51 (46–60)]	[47 (17–56)]	**<0.001** ‡	>0.05	**<0.001**	**0.004**
Central ‡	[19 (10–23)]	[16 (11–42)]	[37 (17–57)]	**<0.001** ‡	>0.05	**<0.001**	**<0.001**
Temporal I	49 ± 2	48 ± 5	35 ± 10	**<0.001**	>0.05	**<0.001**	**<0.001**
Temporal II	35 ± 4	35 ± 3	27 ± 6	**<0.001**	>0.05	**<0.001**	**<0.001**
GCLTQ I [ratio]	0.89 ± 0.04	0.91 ± 0.07	0.78 ± 0.14	**0.001**	0.60	**0.025**	**0.002**
GCLTQ II [ratio]	0.95 ± 0.08	0.93 ± 0.09	0.77 ± 0.17	**<0.001**	0.99	**0.003**	**0.002**

*n*: number; f: female; OD: right eye; SE: spherical equivalent; GCLT: ganglion cell layer thickness; GCLTQ I and II: GCLT quotient of temporal and nasal quadrants within the 3 mm and 6 mm ETDRS rings. Comparisons: healthy (HC) vs. (i) nystagmus without albinism (N_other_) and (ii) nystagmus with albinism (N_albinism_) groups and (iii) N_other_ vs. MSR N_albinism_. ± standard deviation; § Fisher’s exact test; ‡ nonparametric tests: Kruskal Wallis test and data in [median (range)]. Significant *p* values are given in bold.

## Data Availability

Data are available upon request.
